# Complete Mitochondrial Genome of Three Species of the Genus *Microtus* (Arvicolinae, Rodentia)

**DOI:** 10.3390/ani10112130

**Published:** 2020-11-16

**Authors:** Luz Lamelas, Gaël Aleix-Mata, Michail Rovatsos, Juan Alberto Marchal, Teresa Palomeque, Pedro Lorite, Antonio Sánchez

**Affiliations:** 1 Departamento de Biología Experimental, Área de Genética, Universidad de Jaén, Paraje de las Lagunillas s/n, 23071 Jaén, Spain; ldla0001@red.ujaen.es (L.L.); galeix@ujaen.es (G.A.-M.); jamaor@ujaen.es (J.A.M.); tpalome@ujaen.es (T.P.); plorite@ujaen.es (P.L.); 2 Department of Ecology, Faculty of Science, Charles University, 12844 Prague, Czech Republic; michail.rovatsos@natur.cuni.cz

**Keywords:** complete mitogenome, *Microtus*, mitochondrial, phylogeny, control region

## Abstract

**Simple Summary:**

The knowledge and analysis of mitochondrial genomes can be useful to answer some interesting questions about the biology of some species. This is the case of vole species that are characterized by unusual genetic features related to sex chromosomes and variation of their karyotypes. In this work, we describe the mitochondrial genomes of three emblematic vole species, demonstrating that they are highly conserved in the organization of their protein coding, transfer RNA and ribosomal RNA genes. In addition, we performed a detailed analysis of their control regions identifying several domains and conserved boxes related to mitochondrial DNA regulation. Finally, a phylogenetic analysis with the mitochondrial DNA confirmed the established phylogeny of the analyzed voles.

**Abstract:**

The 65 species of the genus *Microtus* have unusual sex-related genetic features and a high rate of karyotype variation. However, only nine complete mitogenomes for these species are currently available. We describe the complete mitogenome sequences of three *Microtus*, which vary in length from 16,295 bp to 16,331 bp, contain 13 protein-coding genes (PCGs), two ribosomal RNA genes, 22 transfer RNA genes and a control region. The length of the 13 PCGs and the coded proteins is the same in all three species, and the start and stop codons are conserved. The non-coding regions include the L-strand origin of replication, with the same sequence of 35 bp, and the control region, which varies between 896 bp and 930 bp in length. The control region includes three domains (Domains I, II and III) with extended termination-associated sequences (ETAS-1 and ETAS-2) in Domain I. Domain II and Domain III include five (CSB-B, C, D, E and F) and three (CSB-1, CSB-2, and CSB-3) conserved sequence blocks, respectively. Phylogenetic reconstructions using the mitochondrial genomes of all the available *Microtus* species and one representative species from another genus of the Arvicolinae subfamily reproduced the established phylogenetic relationships for all the Arvicolinae genera that were analyzed.

## 1. Introduction

The rodent subfamily Arvicolinae (lemmings, muskrats and voles) includes numerous species that have been classified into 21 genera [1], of which the genus *Microtus* is the most species-rich (65 extant species). This genus underwent fast recent radiation about 1.2–2 million years ago (MYA) [2] in what was one of the most explosive speciation events ever recorded in vertebrates [1].

Some species from the genus *Microtus* are characterized by a series of unusual sex-related genetic features [3,4,5,6,7,8,9,10]. Moreover, the genus *Microtus* shows one of the highest rates of karyotype variation of all mammals [11], with highly variable chromosome numbers ranging from 2n = 18 in *M. oregoni* [12] to 2n = 62 in *M. duodecimcostatus* and *M. lusitanicus* [13]. Despite this variability in their karyotypes, the majority of *Microtus* species have similar external morphologies and therefore are hard to distinguish using traditional taxonomic characteristics [14,15,16,17]. In addition, the genus *Microtus* underwent a recent rapid radiation resulting in the emergence of numerous cryptic species with unclear phylogenetic relationships [18,19,20,21]. Although some of these cryptic species can be distinguished using mitochondrial and/or nuclear markers [22,23], *Microtus* phylogeny is not clearly established and many systematic relationships within this genus are still unresolved [20,24].

Phylogenetic reconstructions have been carried out using karyotype banding, chromosome painting and repeated DNA sequence analysis [3,8,25,26]. Despite lacking the reliability to solve intra-specific relationships [27], mitochondrial markers COI and NADH are nevertheless regarded as very precise tools for deciphering interspecific relationships between Arvicolinae species [16,17,24,28]. Several nuclear genetic markers such as the growth hormone receptor (GRH) including exon 10 [27] have been employed to solve intra-specific relationships in the genus *Microtus.* An interesting phylogenomic approach employing partial mitogenomes and genotyping-by-sequencing single nucleotide polymorphism (SNP) data from seven *Microtus* species has also been recently described [29].

Traditionally, complete mitogenomes have been sequenced by PCR amplification of overlapping fragments [30,31]. However, next-generation sequencing (NGS) now allows us to assemble much more easily the whole mitochondrial genome due to its high-copy portion in relation to the nuclear DNA [32,33,34,35,36,37]. Nevertheless, although complete mitochondrial genomes are very useful for phylogenetic reconstructions, the number of species with fully described mitogenomes is still low [38,39]. Only eight complete mitogenomes are currently available for *Microtus* species, and therefore, in light of the fast radiation processes taking place in this genus, the description of mitogenomes from other *Microtus* species is potentially of great interest. In this paper, we report the sequencing and characterization by next-generation sequencing (NGS) data of the complete mitogenome of three further *Microtus* species, *M. cabrerae, M. chrotorrhinus* and *M. thomasi*.

## 2. Materials and Methods

### 2.1. DNA Extraction, Sequencing and Mitogenome Assembly

A single male specimen of *M. cabrerae* (Siles, Spain (TOPC-0902)) and one of *M. thomasi* (Edessa, Greece, (R231)) were used for DNA extraction, while for *M. chrotorrhinus* (North America), an established cell line from a male individual was employed [4,40]. All capture and sacrifice protocols were approved by the Junta de Andalucía Ethics Committee for Animal Experimentation (code: 04/09/2018/130). Tissue samples from the specimens were stored in 100% ethanol at −20 °C. The total DNA was extracted from tissues and from cultured cells using the Gentra Puregene Tissue Kit (Qiagen, Hilden, Germany). The extracted genomic DNA of *M. cabrerae* was sequenced using the Illumina technology in Macrogen (Europe) and *M. thomasi* and *M. chrotorrhinus* DNAs in Novogene (Hong Kong). Briefly, for genome sequencing, we used approximately 3 µg of genomic DNA for the construction of a library of 750-bp-long fragments for *M. cabrerae* and 350-bp-long fragments for the other two species. These libraries were sequenced using the Illumina^®^ Hiseq™ 2000 (San Diego, CA, USA) platform with the 2 × 100 nt paired-end option. Two Gbp of sequences were obtained for each species. Graph-based clustering analysis [41,42] was performed using RepeatExplorer implemented within the Galaxy environment (http://repeatexplorer.org/). Sequence clusters corresponding to mtDNA were selected and assembled. We also used the Geneious v.R7.1 software [43] to assemble and annotate the mitogenomes. Both assembled versions of each species’ mitogenome were aligned and revised manually using the complete *M. arvalis* mitogenome as reference [38] (accession number: MG948434). In addition, the sequence of the control region (D-loop) of the mitochondrial genomes was confirmed by PCR amplification and sequencing using the primer pair Pro+ (5′-ACCATCAGCACCCAAAGCTG-3′) and Phe- (5′-AAGCATTTTCAGTGCTTTGCTT-3′) [35,44].

### 2.2. Annotation and Sequence Analysis

The annotation of these three mitogenomes was performed using web-based services MITOS (http://mitos.bioinf.uni-leipzig.de/help.py) [45] and tRNA scan-SE (http://lowelab.ucsc.edu/tRNAscan-SE/) [46]. The annotations of the protein-coding genes (PCGs), transfer RNAs (tRNAs) and rRNA genes were refined by manual comparison, according to Markova et al. [47], with the *M. arvalis* mitogenome [38]. The base composition was estimated using the Bioedit program (v7.0.9.0) (http://www.mbio.ncsu.edu/BioEdit/bioedit.html), and codon usage was analyzed using MEGA version 10 [48]. The circularized drawing of the mitogenome was performed with the OrganellarGenomeDRAW tools (http://ogdraw.mpimp-golm.mpg.de/) [49].

### 2.3. Phylogenetic Analysis

For the phylogenetic analyses, in addition to the sequenced mitogenomes described above, we also included nine complete mitochondrial genomes from *Microtus* species available in GenBank and from other representative species of the Arvicolinae subfamily [1,38,50,51,52,53,54,55,56,57,58,59,60,61,62]. As outgroup, we used a species of the genus *Akodon* (*A. montensis*) [63] from the subfamily Sigmodontinae.

Complete mitogenomes were aligned using ClustalW, and the phylogenetic relationships were reconstructed using the Bayesian inference (BI) implemented in *MrBayes* v. 3.1 [64]. Runs of two million generations were conducted. Trees were sampled every 1000 generations with a burn-in of 25%. The best-fit nucleotide substitution model with the lowest BIC (Bayesian Information Criterion) value was chosen (GTR + G + I) using MEGA version X [48].

## 3. Results and discussion

### 3.1. Gene Organization

The complete mitogenomes of *M. cabrerae* (MN058077), *M. chrotorrhinus* (MN058078) and *M. thomasi* (MN058079) analyzed were 16,331 bp, 16,297 bp and 16,295 bp in length, respectively. These values are similar to the mitogenomes of other species from this genus, which range between 16,283 bp (*M. rossiaemeridionalis*) and 16,312 bp (*M. kikuchii*) [1,60]. They were also comparable in size to those from other species of the Arvicolinae subfamily such as *Proedromys liangshanensis* (16,296 bp) [57] and *Neodon forresti* (16,397 bp; GenBank accession number: KU891252.1). All our results confirm that the mitochondrial genomes in the Arvicolinae subfamily are very similar in size.

**Figure 1:**
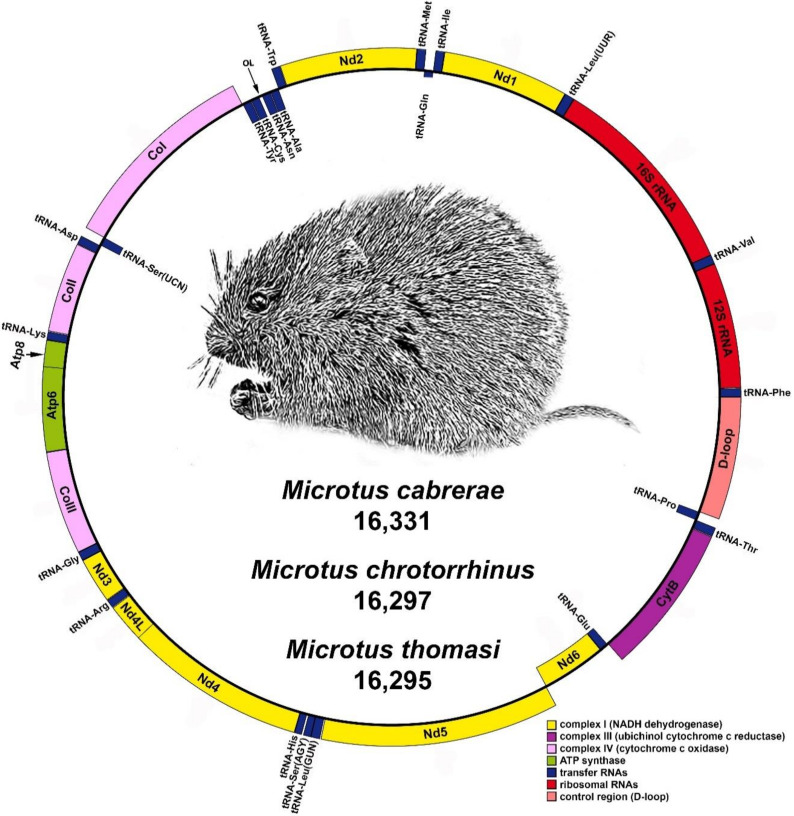
Representative map of the mitochondrial genome of the three analyzed *Microtus* species. Genes encoded by the heavy strand are shown outside the circle, while those encoded by the light strand are shown inside.

The mitogenomes from *M. cabrerae*, *M. chrotorrhinus* and *M. thomasi* include a control region (D-loop) and a conserved set of 37 vertebrate mitochondrial genes, with 13 protein-coding genes (PCGs), 22 tRNA genes and two rRNA genes (*12S rRNA* and *16S rRNA*) (Table 1). As expected, the organization and structures of these three mitogenomes were identical to those described for other *Microtus* and mammal species (Figure 1). Hence, twelve PCGs, 14 *tRNAs* and two *rRNAs* are located on the heavy strand, while *Nd6* and eight *tRNAs* are found on the light strand. The D-loop is emplaced between the *tRNA-Pro* and *tRNA-Phe* genes [37,38,57]. The percentage of identity observed in pairwise comparing of these three complete mitogenomes varied between 86.34% (*M. thomasi–M. cabrerae* pair-wise comparison) and 87.55% (*M. thomasi–M. chrotorrhinus* pair-wise comparison). These results fall into the range of the identity values that we calculated for comparisons between the available *Microtus* mitogenomes (85.50–98.75%).

### 3.2. Nucleotide Composition

We identified a bias towards A and T nucleotides, which is commonly reported in mitogenome sequences in mammals [35,37]. Hence, the A+T compositions of the H-strands are 58.19%, 59.27% and 59.77% in the *M. cabrerae*, *M. chrotorrhinus* and *M. thomasi* mitogenomes, respectively. The 13 mitochondrial PCGs are AT-biased, with an A+T content ranging from 54.34–56.63% for *Cox3* to 64.71–66.67% for the *Atp8* gene. The control region, the two rRNA genes and the 22 tRNAs are also AT-biased in all three species (Table 2).

### 3.3. Protein-Coding Genes and Codon Usage

The 13 mitochondrial PCGs from the three analyzed species are 11,390 bp in length (11,358 bp in codons and 32 bp in stop codons) and encode 3786 amino acids (Table 2). The three described mitogenomes also contain some overlapping nucleotides and gaps between PCGs or between PCGs and tRNAs (Table 1). The longest overlap of 43 bp is located between the *Atp8* and *Atp6* genes (Table 1).

Twelve mitochondrial PCGs use exactly the same start codon for translation initiation in all the three species: GTG for *Nd1*, ATT for *Nd2* and *Nd3*, ATG for the other nine PCGs. Only the Nd5 gene has variation in the start codon: ATA in *M. cabrerae* and *M. thomasi*, and ATT in *M. chrotorrhinus*. Similarly, 12 PCGs genes use exactly the same five stop codons for translation termination in all three species, two incomplete (T-- for *Nd1, Cox3* and *Nd4*; TA- for *Atp6*) and the three other complete (TAG for *Nd6*, TAA for the rest of PCGs). However, Nd5 uses TAA in *M. cabrerae* and *M. thomasi*, and TAG in *M. chrotorrhinus* (Table 2).

The most abundant start and stop codons were ATG and TAA, respectively, a finding that agrees with previous work on other mammal mitogenomes [30,31,35,37,65,66]. Incomplete stop codons (T-- or TA-), like those used in three PCGs (*Nd1*, *Cox3*, and *Nd4*), are commonly observed in metazoan mitogenomes. They might be further completed by poly-adenylation of the 3′-end of the mRNA occurring after transcription, giving rise to the complete functional TAA stop codon [67,68].

The length of the 13 PCGs is the same in all three species and, consequently, in the coded proteins as well. The percentages of nucleotide identity range between 82.03%, observed when comparing the *M. cabrereae* and *M. thomasi Nd2* gene sequences, and 89.71%, obtained when comparing the *M. chrotorrhinus* and *M. thomasi Atp8* gene sequences (Table 2).

Due to the A+T richness of the *Microtus* mitogenomes, a strong bias toward A+T-rich codons in the codon usage of the PCGs was observed (Table 3). The most frequently used codons are CTA (Leu), ATC (Ile), ATA (Met), ATT (Ile), TTC (Phe), ACA (Thr), AAC (Asn), TCA (Ser), CCA (Pro) and GCC (Ala) (Table 3). Accordingly, leucine (15.1–15.5%), isoleucine (8.9–9.7%), threonine (8.2–8.3%), serine (7.5–7.7%), alanine (6.8–6.9%), phenylalanine (6.2–6.3%), methionine (5.7–6.0%) and glycine (5.6–5.7%) are the most common amino acids found in the mitochondria protein sequences, comprising approximately 65% of the coded amino acids. The high proportion of A+T in PCGs appears to be a shared feature in mammal species [35,66]. For any amino acid, the relative synonymous codon usage value (RSCU) is equivalent to the number of times that a codon appears in a gene in relation to the number of expected occurrences under an assumption of equal codon usage. The six codons with the highest RSCU values described in the PCGs from *Microtus* are as follows: CTA(L) (2.58–2.62), TCA(S) (2.40–2.55), CGA(R) (2.38–2.50), ACA(T) (1.97–2.01), CCA(P) (1.95–2.22) and GCC(A) (1.81–2.05), three of which are A+T rich (Table 3).

### 3.4. rRNAs, tRNA Genes and Non-Coding Regions

The *tRNA-Val* is located between the rRNA genes *12S* and *16S*. rRNAs genes appeared flanked by *tRNA-Phe* and *tRNA-Leu(UUR)* (Table 1; Figure 1). The combined sizes of the two rRNA genes in *M. cabrerae*, *M. chrotorrhinus* and *M. thomasi* are 2518 bp, 2509 bp and 2515 bp, respectively. The combined sizes of the 22 tRNA genes in *M. cabrerae*, *M. chrotorrhinus* and *M. thomasi* are 1488 bp, 1500 bp and 1489 bp, respectively. The length of the tRNA genes ranged between 56 bp for *tRNA-Hys* in *M. cabrerae* to 75 bp for *tRNA-Leu(UUR)* in the three mitogenomes (Table 1).

**Figure 2:**
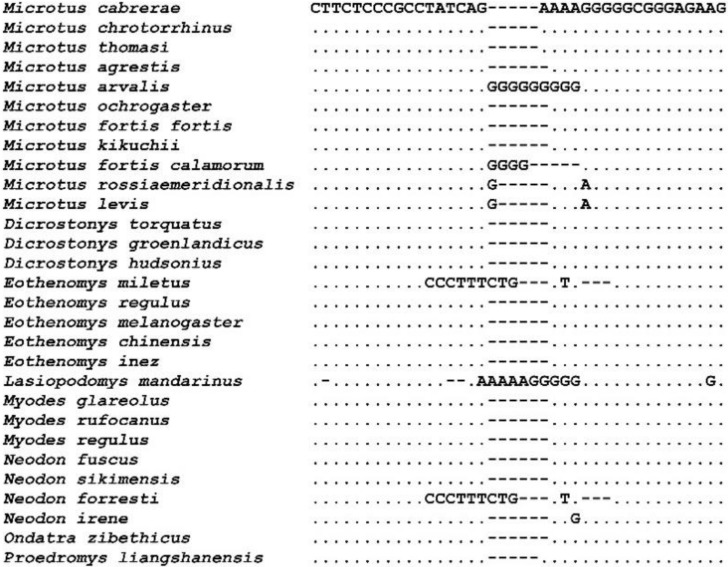
Nucleotide sequence alignment of the L-strand origin of replication (*O_L_*) of species from the subfamily Arvicolinae. Dots indicate identity and dashes denote deletions.

Non-coding regions are important during replication and for the maintenance of the mitogenomes. These included the L-strand origin of replication (*OL*), intergenic spacers and the control region [69]. The three mitogenomes from *Microtus* species analyzed here have identical ORs of 35 bp. This region is located between *tRNA-Asn* and *tRNA-Cys* in the WANCY region, which refers to a cluster of five tRNA genes (*tRNA-Trp*, *tRNA-Ala*, *tRNA-Asn*, *tRNA-Cys* and *tRNA-Tyr*). The same organization is present in other *Microtus* and Arvicolinae species, as well as in most mammal species [31,37,38,57]. Hence, the OR from the mitogenomes of other *Microtus* species varies in length from 34 to 40 bp, while in most Arvicolinae species it is 34 bp in length and is highly conserved (Figure 2). Intergenic spacers were also found in the mitogenomes, with sizes in the range 1–11 bp (Table 1).

The D-loops in *M. cabrerae*, *M. chrotorrhinus* and *M. thomasi* are 930 bp, 903 bp and 896 bp in length, respectively, and are located between *tRNA-Pro* and *tRNA-Phe* (Table 1; Figure 1). In other Arvicolinae species, the length of this region ranges between 657 bp (*M. ochrogaster)* and 1089 bp (*Myodes rufocanus*). Control regions are the most clearly differentiated regions in the mitogenomes of the three *Microtus* species analyzed, with a nucleotide similarity of 80.67–82.09%. Similar values are observed when comparing the D-loop regions of different *Microtus* species (77.1–97.3%) and those from other Arvicolinae species (64.8–97.8%).

**Figure 3:**
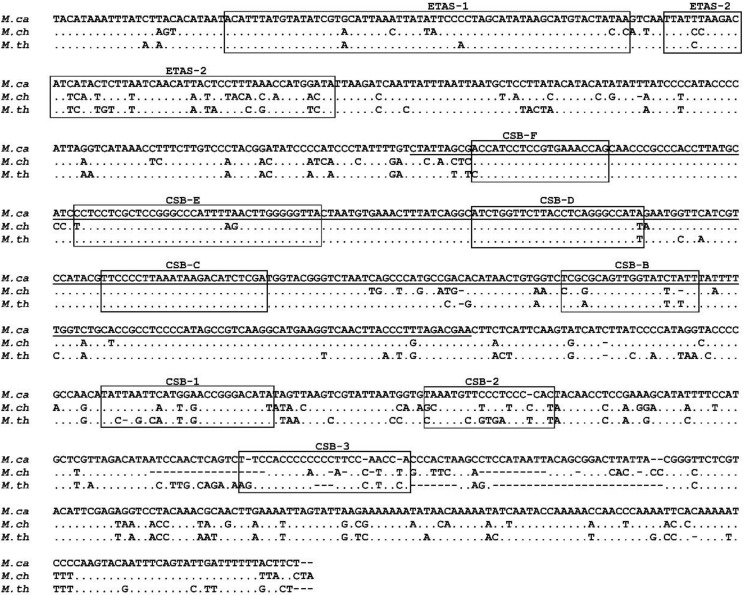
Nucleotide sequence alignment of the control region of *Microtus cabrerae* (*M. ca*), *M. chrotorrhinus* (*M. ch*) and *M. thomasi* (*M. th*). Dots indicate the identity of the nucleotides and dashes the indels. All the conserved sequences identified are included in boxes: the two conserved extended termination-associated sequences (ETAS1 and ETAS2) in Domain I, five conserved blocks (CSB-F, E, D, C, B) in the central conserved Domain II (underlined) and three conserved blocks (CSB1, CSB-2 and CSB-3) in Domain III. Dots indicate identity and dashes denote deletions.

The control region includes three domains (Domains I, II and III). In Domain I, the extended termination-associated sequences (ETAS-1 and ETAS-2) were identified (Figure 3). The ETAS-1 sequence is better conserved than the ETAS-2 sequences in these species. Thus, pair-wise comparisons of ETAS-1 sequences showed a similarity of 89.83–96.6%, while the ETAS-2 sequence similarity was 67.3–76.9%.

The conserved sequence blocks CSB-1, CSB-2 and CSB-3 [70,71] were identified within Domain III. CSB-1 is the best-conserved block, having similarity values of 66.7–84% when comparing the three *Microtus* species with each other. CSB-2 and CSB-3 are less well conserved, with similarities of 52.6–68.4% and 54.1–68.0%, respectively (Figure 3). No repetitive DNA sequences were found to be present between CSB1 and CSB2 on the *Microtus* D-loop, as occurs in other mammal species [35]. Five other conserved sequence blocks (CSB-B, C, D, E and F) were identified in central Domain II [72], all of which are well conserved with nucleotide similarities of 78.95–100% (Figure 3).

### 3.5. Phylogenetic Analysis

**Figure 4:**
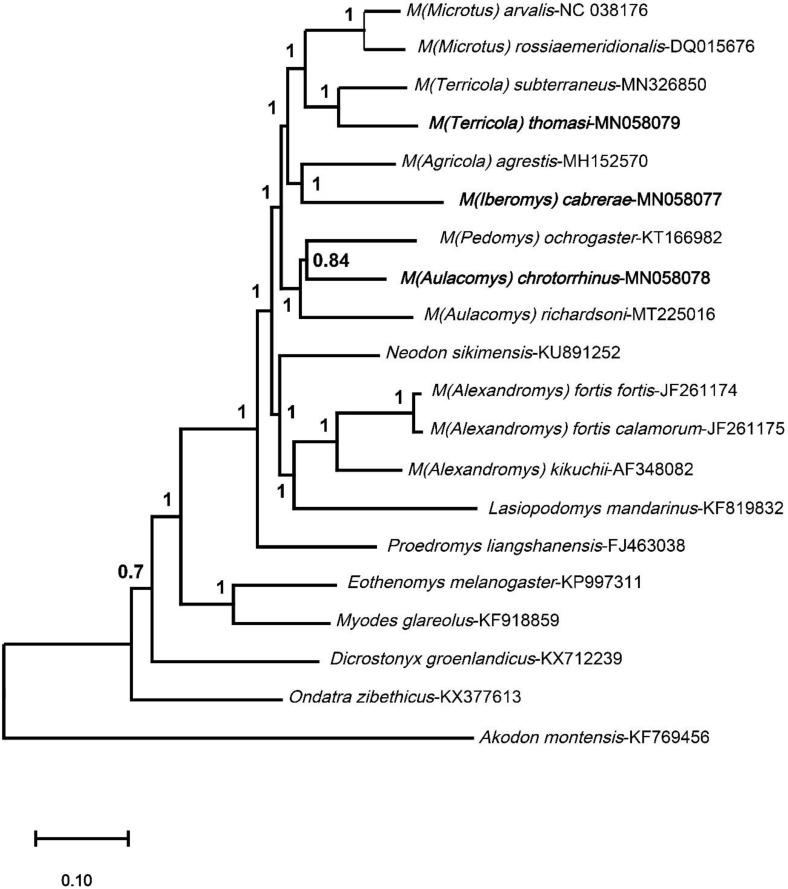
Bayesian Inference tree using the complete mitogenome sequences of ten *Microtus* species available in GenBank and seven representative species of genera from the subfamily Arvicolinae, and the mitogenome sequence of *Akodon montensis* (subfamily Sigmodontinae) used as an outgroup. Bayesian posterior probabilities are indicated at each node.

The phylogenetic positions of the three analyzed species were assessed using Bayesian inference (Figure 4). All *Microtus* species are clustered in a well-supported clade that also includes *Neodon* and *Lasiopodomys* species. The *Alexandromys* species are grouped in the same branch with genera *Neodon* and *Lasiopodomys,* which have been considered as subgenera of *Microtus* genus, although phylogenetic relationships are not clearly established [20,24]. *Proedromys* is also close to this *Microtus* clade since it appears grouped as part of a well-supported node (posterior probability values = 1). The *Microtus-Proedromys* group is also associated with the clade of *Myodes* and *Eothenomys* (posterior probability values = 0.93). Finally, *Dicrostonyx* and *Ondatra* show a basal position. These results agree with the previously established phylogenetic relationships for these genera [73,74,75,76].

A number of conclusions can be drawn for the *Microtus* species analyzed. The two species from the *Terricola* group included here, *M. (Terricola) thomasi* and *M. (Terricola) subterraneus*, are closely associated with *M. arvalis* and *M. rossiaemeridionalis*. The close phylogenetic relationship between the *Terricola* and *Microtus* subgenera has been previously reported [20,29]. Although the two subgenera *Aulacomys* and *Pedomys* are not resolved the three North American species, *M. chrotorrhinus, M. ochrogaster* and *M. richardsoni* are grouped together. These Nearctic species fall within the phylogeny of *Microtus,* in line with previous studies [20], rather than being basal, as recently reported [29]. *M. cabrerae* and *M. agrestis* are grouped, which could support their inclusion in the subgenus *Agricola* as has been previously proposed [20]. Ostensibly, *M. cabrerae* and *M. agrestis* share certain unusual genetic features, including the presence of giant sex chromosomes, which could be regarded as additional proof of their phylogenetic proximity. However, several studies have clearly demonstrated that these enlarged sex chromosomes arose and evolved independently in the genus *Microtus* [4], and hence, their presence is not a robust criterion for the inclusion of *M. cabrerae* and *M. agrestis* in the same subgenus. A previous mitochondrial phylogenetic reconstruction obtained similar results, with *M. cabrerae* and *M. agrestis* clustered in the same clade, but the level of genetic divergence indicated that both species could be considered as members of two different subgenera (*Agricola* and *Iberomys*) [29]. The genus *Iberomys*, which is based on the description of archaic morphological characters, has been proposed, with only the species *Microtus cabrerae* [77,78]; however, no support is obtained for this genus [20].

The phylogenetic results obtained here with mitochondrial data need to be validated by the use of other nuclear markers because of the limitation inherent to mitogenomes, maternal inheritance, accelerated rates of substitution, introgression, effective population size and neutrality [79]. However, sequencing and characterization of mitogenomes from other species of the genus *Microtus* and closely related taxa from the subfamily Arvicolinae will help our understanding of the phylogenic relationships of this rodent species group and hence resolve some of the issues that remain open.

## 4. Conclusions

The complete mitogenomes of three *Microtus* species are described. Our results demonstrated that these mitogenomes have the organization and characteristics of the described mitogenomes of voles and mammalian species and contain 13 protein-coding genes (PCGs), two ribosomal RNA genes, 22 transfer RNA genes and a control region. We identified the conserved domain and the sequence-conserved blocks of the region control. Phylogenetic reconstructions reproduced the established phylogenetic relationships for all the Arvicolinae genera that were analyzed. Our results could be useful in future studies about the identifications and phylogeny of Arvicolidae species, especially of the genus *Microtus*.

## Figures and Tables

**Table 1:** Gene organization of the three *Microtus* mitogenomes (*M.ca*: *M. cabrerae*; *M.ch*: *M. chrotorrhinus*; *M.th*: *M. thomasi*).

Gene	Nucleotide Positions			Size (bp) ^1^			Strand ^2^	Anticodon	Intergenic Nucleotide ^1^		
	*M.ca*	*M.ch*	*M.th*	*M.ca*	*M.ch*	*M.th*			*M.ca M.ch M.th*		
tRNA^Phe^	1–66			66			H	TTC			
12S rRNA	69–1018	69–1015	69–1016	950	947	948	H		2		
tRNA^Val^	1019–1089	1018–1085	1016–1086	71	70	70	H	GTA			
16S rRNA	1088–2655	1084–2645	1085–2651	1568	1562	1567	H		−2		
tRNA^Leu (uur)^	2656–2730	2647–2721	2653–2727	75			H	TTA		1	1
Nd1	2731–3685	2722–3676	2728–3682	955			H				
tRNA^Ile^	3686–3753	3677–3745	3683–3751	68	69	69	H	ATC			
tRNA^Gln^	3751–3822	3743–3814	3749–3820	72			L	CAA	−3		
tRNA^Met^	3824–3892	3817–3885	3822–3890	69			H	ATG	1	2	1
Nd2	3893–4927	3886–4920	3891–4925	1035			H				
tRNA^Trp^	4929–4995	4922–4988	4927–4993	67			H	TGA	1		
tRNA^Ala^	4997–5065	4990–5058	4995–5063	69			L	GCA	1		
tRNA^Asn^	5067–5136	5061–5130	5066–5135	70			L	AAC	1	2	2
Rep_origin	5137–5171	5131–5165	5136–5170		35		H				
tRNA^Cys^	5169–5236	5163–5230	5168–5235	68	69	68	L	TGC	−3		
tRNA^Tyr^	5237–5303	5231–5297	5236–5302	67			L	TAC			
Cox1	5305–6849	5299–6843	5304–6848	1545			H		1		
tRNA^Ser (ucn)^	6847–6915	6841–6909	6846–6914	69			L	TCA	−3		
tRNA^Asp^	6919–6986	6913–6980	6918–6985	68			H	GAC	3		
Cox2	6988–7671	6982–7665	6987–7670	684			H		1		
tRNA^Lys^	7675–7737	7669–7732	7674–7737	63	64	64	H	AAA	3		
Atp8	7738–7941	7733–7936	7738–7941	204			H				
Atp6	7899–8578	7894–8573	7899–8578		680		H		−43		
Cox3	8579–9362	8574–9357	8579–9362	784			H				
tRNA^Gly^	9363–9430	9358–9425	9363–9430	68			H	GGA			
Nd3	9431–9778	9426–9773	9431–9778	348			H				
tRNA^Arg^	9780–9845	9775–9841	9780–9846	66	67	67	H	CGA	1		
Nd4L	9849–10145	9844–10140	9850–10146	297			H		3	2	
Nd4	10139–11516	10134–11511	10140–11517	1378			H		−7		
tRNA^His^	11517–11572	11512–11579	11527–11585	56	68	59	H	CAC			9
tRNA^Ser^	11584–11642	11580–11638	11586–11644	59			H	AGC	11		
tRNA^Leu^	11642–11711	11638–11707	11644–11713	70			H	CTA	−1		
Nd5	11712–13523	11708–13519	11714–13525	1812			H				
Nd6	13523–14044	13516–14040	13522–14046	525			L		−4		
tRNA^Glu^	14045–14113	14041–14109	14047–14115	69			L	GAA			
CytB	14119–15261	14115–15257	14121–15263	1143			H		5		
tRNA^Thr^	15264–15333	15260–15326	15266–15331	70	67	66	H	ACA	2		
tRNA^Pro^	15334–15401	15327–15394	15332–15399	68			L	CCA			
D-loop	15402–16331	15395–16297	15400–16295	930	903	896	H				

^1^ The corresponding three values are only indicated when they are different. ^2^ Strand: H: heavy and L: light.

**Table 2:** Data about the 13 PCG of the three *Microtus* mitogenomes (*M.ca*: *M. cabrerae*; *M.ch*: *M. chrotorrhinus*; *M.th*: *M. thomasi*).

Gene	Gene Length (bp)	% Identity			A + T Content (%)			Start/Stopcodons	Protein Length (aa)
		*M.ca/M.ch*	*M.ca/M.th*	*M.ch/M.th*	*M.ca*	*M.ch*	*M.th*	*M.ca/M.ch/M.th*	
*Nd1*	955	84.50	85.86	86.39	56.96	57.49	57.70	GTG/T--	318
*Nd2*	1035	82.22	82.03	83.96	59.32	61.84	61.93	ATT/TAA	344
*Cox1*	1545	86.21	86.15	86.28	55.34	57.86	56.50	ATG/TAA	514
*Cox2*	684	86.11	86.11	85.38	56.29	57.46	58.33	ATG/TAA	227
*Atp8*	204	85.78	87.75	89.71	64.71	66.67	65.69	ATG/TAA	67
*Atp6*	680	86.93	84.58	86.78	56.39	56.98	59.77	ATG/TA-	226
*Cox3*	784	85.33	84.44	85.84	54.34	56.63	56.12	ATG/T--	261
*Nd3*	348	83.33	83.33	84.48	58.62	57.76	59.48	ATT/TAA	115
*Nd4L*	297	82.83	82.83	82.49	57.91	55.89	59.60	ATG/TAA	98
*Nd4*	1378	82.15	80.48	83.74	57.84	58.35	60.16	ATG/T--	459
*Nd5*	1812	83.17	82.34	85.10	56.29	57.73	58.44	ATA/TAA ATT/TAG ATA/TAA	603
*Nd6*	525	85.14	85.52	87.62	60.76	63.05	60.76	ATG/TAG	174
*CytB*	1143	87.66	87.75	88.10	56.52	56.26	58.53	ATG/TAA	380
**Total**	11,390								3786

**Table 3:** Codon usage of mitochondrial genomes protein-coding genes (PCG) of the three *Microtus* species.

Codon	n	%	RSCU	Codon	n	%	RSCU
UUU(F)	69–92	1.8–2.4	0.58–0.79	UAU(Y)	53–56	1.4–1.5	0.87–0.91
UUC(F)	142–169	3.7–4.5	1.21–1.42	UAC(Y)	65–69	1.7–1.8	1.09–1.13
UUA(L)	84–105	2.2–2.8	0.86–1.10	UAA(*)	11–12	0.3	3.38–3.69
UUG(L)	15–20	0.4–0.5	0.15–0.20	UAG(*)	1–2	0–0.1	0.31–0.62
CUU(L)	82–90	2.2–2.4	0.85–0.92	CAU(H)	13–30	0.3–0.8	0.26–0.61
CUC(L)	79–100	2.1–2.6	0.82–1.02	CAC(H)	68–87	1.8–2.3	1.39–1.74
CUA(L)	251–254	6.6–6.7	2.58–2.62	CAA(Q)	66–67	1.7–1.8	1.72–1.74
CUG(L)	35–46	0.9–1.2	0.36–0.47	CAG(Q)	10–11	0.3	0.26–0.28
AUU(I)	142–178	3.7–4.7	0.77–1.00	AAU(N)	35–43	0.9–1.1	0.43–0.52
AUC(I)	178–229	4.7–6.0	1.00–1.24	AAC(N)	122–130	3.2–3.4	1.48–1.57
AUA(M)	164–173	4.3–4.6	1.48–1.59	AAA(K)	83–94	2.2–2.5	1.66–1.86
AUG(M)	45–60	1.2–1.6	0.41–0.53	AAG(K)	7–17	0.2–0.4	0.14–34
GUU(V)	22–33	0.6–0.9	0.54–0.73	GAU(D)	20–26	0.5–0.7	0.53–0.70
GUC(V)	46–50	1.2–1.3	1.01–1.22	GAC(D)	48–55	1.3–1.4	1.30–1.47
GUA(V)	73–86	1.9–2.3	1.74–1.82	GAA(E)	65–83	1.7–2.2	1.38–1.75
GUG(V)	19–24	0.5–0.6	0.46–0.53	GAG(E)	12–29	0.3–0.8	0.25–0.62
UCU(S)	37–38	1	0.76–0.80	UGU(C)	6–8	0.2	0.38–0.50
UCC(S)	62–76	1.6–2.0	1.30–1.56	UGC(C)	24–26	0.6–0.7	1.50–1.63
UCA(S)	117–122	3.1–3.2	2.40–2.55	UGA(W)	75–96	2–2.5	1.47–1.88
UCG(S)	6–12	0.2–0.3	0.13–0.25	UGG(W)	6–27	0.2–0.7	0.12–0.53
CCU(P)	33–50	0.9–1.3	0.64–1.01	CGU(R)	4–9	0.1–0.2	0.25–0.56
CCC(P)	34–56	0.9–1.5	0.68–1.09	CGC(R)	10–19	0.3–0.5	0.63–1.19
CCA(P)	99–114	2.6–3.0	1.95–2.22	CGA(R)	38–40	1–1.1	2.38–2.50
CCG(P)	2–17	0.1–0.4	0.04–0.33	CGG(R)	3–5	0.1	0.19–31
ACU(T)	55–58	1.4–1.5	0.69–0.75	AGU(S)	12–17	0.3–0.4	0.25–0.36
ACC(T)	87–94	2.3–2.5	1.12–1.19	AGC(S)	36–43	0.9–1.1	0.75–0.88
ACA(T)	153–159	4.0–4.2	1.97–2.01	AGA(*)	0	0	0
ACG(T)	7–12	0.2–0.3	0.09–0.15	AGG(*)	0	0	0
GCU(A)	39–52	1.0–1.4	0.60–0.80	GGU(G)	37–41	1–1.1	0.69–0.75
GCC(A)	116–134	3.1–3.5	1.81–2.05	GGC(G)	50–65	1.3–1.7	0.93–1.19
GCA(A)	78–82	2.1–2.2	1.19–1.28	GGA(G)	58–86	1.5–2.3	1.06–1.61
GCG(A)	8–11	0.2–0.3	0.12–0.17	GGG(G)	41–54	1.1–1.4	0.75–0.99

RSCU: relative synonymous codon usage. * Termination codon.
